# Interaction between *crgA* and *wc-1b* in carotenoid synthesis in *Blakeslea trispora*

**DOI:** 10.3389/fbioe.2026.1838537

**Published:** 2026-07-28

**Authors:** Youai Dai, Renyue Gui, Jingyu Chen, Wei Luo

**Affiliations:** 1 Wuxi School of Medicine, Jiangnan University, Wuxi, China; 2 The Affiliated Wuxi People’s Hospital of Nanjing Medical University, Wuxi People’s Hospital, Wuxi Medical Center, Nanjing Medical University, Wuxi, Jiangsu, China; 3 The Key Laboratory of Industrial Biotechnology, Ministry of Education, School of Biotechnology, Jiangnan University, Wuxi, China

**Keywords:** *Blakeslea trispora*, BtCrgA, BtWC-1b, protein interaction, RNA interference

## Abstract

β-Carotene is an important pigment widely used in the food industry, and its large-scale production can be achieved using *Blakeslea trispora*. In *B*. *trispora*, CrgA is a key negative regulator of β-carotene synthesis, while Wc-1b is a photoreceptor whose role in carotenoid synthesis remains unknown. Although the light-induced synthesis of carotenoids may be related to both, the relationship between them is unclear. To clarify the roles of *crgA* and *wc-1b* in regulating β-carotene synthesis in *B. trispora* and their potential interaction, the effects of *btcrgA* and *btwc-1b* on β-carotene production and the relationship between them were investigated at both the transcriptional and protein levels. The relative transcription levels of *btcrgA, btwc-1b*, and carotenoid structural genes (*carRA* and *carB*) were highly correlated under short-term light irradiation, suggesting that *btcrgA* and *btwc-1b* may interact in regulating β-carotene synthesis. In darkness, *btcrgA* affected β-carotene synthesis by negatively regulating the transcription levels of *carRA* and *carB*, and light attenuated this transcriptional regulation. However, *btcrgA* did not regulate *btwc-1b* at the transcriptional level. Conversely, *btwc-1b* positively regulated the transcription of *carRA* and *carB* and β-carotene synthesis in darkness, but this regulation was completely abolished by light. Likewise, *btwc-1b* did not regulate *btcrgA* at the transcriptional level. At the protein level, BtCrgA and BtWC-1b were shown to interact with each other both *in vitro* and *in vivo*, indicating that BtCrgA may regulate carotenoid synthesis through BtWC-1b.

## 1 Introduction

β-Carotene is a fat-soluble orange-red pigment and a tetraterpenoid compound that is prone to oxidation and sensitive to light and heat. As a natural colorant, antioxidant, and vitamin A precursor, it is widely used in foods, beverages, and dietary supplements. The industrial production of β-carotene primarily utilizes submerged fermentation of *Blakeslea trispora*, a fungus characterized by high yield and low cost, with fed-batch fermentation significantly enhancing production. However, key regulatory factors for β-carotene synthesis in this fungus, such as CrgA and White Collar-1 (WC-1), remain to be fully elucidated. Although the *crgA* gene is widely distributed in a variety of filamentous fungi, only that from the zygomycete *Mucor circinelloides* has been investigated in detail so far. In this fungus, the expression of the *crgA* gene itself is activated by light pulses, and its transcript accumulation is similar to that of other light-induced genes (Navarro et al., 2001). CrgA acts as a repressor of carotenoid biosynthesis, as its deletion leads to enhanced transcript levels of carotenoid structural genes and overaccumulation of carotenoids under both dark and light conditions (Zhang et al., 2016). This protein is characterized by two RING finger domains and a LON domain near the N-terminus (Quiles-Rosillo et al., 2005; Deshaies and Joazeiro, 2009; Tagua et al., 2020). The RING zinc finger domain is a common structural element of the ubiquitin ligase family, which links ubiquitin residues to target proteins.

Molecular genetic investigation of *white collar-1* in *M. circinelloides* (*mcwc-1*) indicates that it is a representative example of subfunctionalization through evolution (Ballario et al., 1996; Cheng et al., 2002; He et al., 2005). The gene is duplicated during an evolutionary event, and each copy appears to have acquired a specific function. Genes such as *mcwc-1a* and *mcwc-1c* may have retained or acquired the ability to perceive light, similar to *wc-1* in *Neurospora crassa* and other fungi. Although both proteins sense light, they control different physiological responses, suggesting that they act on different targets selected through evolution. In some studies, the gene sequences of BtWC-1a, BtWC-1b, and BtWC-1c from *B. trispora* were introduced to the corresponding defective strains of *M. circinelloides*, and the phenotype of the engineered strain was restored (Silva et al., 2006). This shows that the function of these photoreceptors is extremely conserved in these two strains, while their function in *B. trispora* has not been investigated in detail (Aguilera et al., 2000; Luo et al., 2020a).

Protein ubiquitination plays an important role in the regulation of many cellular processes by altering the stability, localization or function of target proteins. CrgA is an ubiquitin ligase-like protein involved in the regulation of carotenoid synthesis and asexual sporulation in *M. circinelloides*. CrgA may reduce the activity of White Collar-1b (WC-1b) through non-proteolytic mono- and di-ubiquitination, thereby activating carotenoid synthesis. When McWC-1b is inactivated by ubiquitination, it forms a conserved Rossmann-fold NAD(P)H/NAD(P)(+) binding domain similar to NmrA NADP(H) (Navarro et al., 2013).

Previous studies in our laboratory have shown that *btcrgA* in *B. trispora* is a negative regulator of β-carotene synthesis (Luo et al., 2020b), but the signal transduction process regulating light-induced carotenoid synthesis is still unclear. This study investigated the interaction between *btcrgA* and *btwc-1b* at the transcriptional and protein levels to infer their possible impact on carotenoid synthesis. The aim was to better understand the signal transduction pathway and molecular mechanism by which *btcrgA* regulates light-induced β-carotene synthesis in *B. trispora*.

## 2 Materials and methods

### 2.1 Strains, plasmids, reagents and media

*Blakeslea trispora* NRRL2896, *Escherichia coli* DH5α and *Agrobacterium tumefaciens* LBA4404 were purchased from Coollab Technology Co., Ltd. Plasmids pCAMBIA1303, pUC57, pBARGPE1-mCherry, mU6-sgRNA and mVenus N1 were obtained from our laboratory stock. Primer synthesis and gene sequencing were provided by Nanjing Genscript Biotechnology Co., Ltd.

The Plant Genomic DNA Kit, Spin Column Fungal Total RNA Purification Kit, Bradford Protein Concentration Assay Kit, GUS Reporter Gene Quantitative Detection Kit, Prime STAR ®Max DNA Polymerase, 2×Rapid Taq Master Mix, Hifair®III 1st Strand cDNA Synthesis Super Mix for qPCR (gDNA digester plus), Hieff ® qPCR SYBR Green Master Mix, Hieff Clone® Plus Multi One Step Cloning Kit, Universal DNA Purification Kit, DNA Small Extraction Kit, Helicase, Cellulase, and Lysozyme, Kanamycin Sulfate, Rifampicin, Acetosyringone, Bradford Protein Concentration Assay Kit, SDS-PAGE Gel Preparation Kit, Coomassie brilliant blue R250, G250, nucleic acid protein Maker, 4′,6-diamidino-2-phenylindole (DAPI) staining solution, horseradish peroxidase-labeled goat anti-rabbit IgG (H + L), protease inhibitor mixture (general type), horseradish peroxidase-labeled goat anti-mouse IgG (H + L), anti-fluorescence quenching mounting solution, Flag antibody (mouse monoclonal antibody), Myc antibody (mouse monoclonal antibody), His antibody (mouse monoclonal antibody), and GST antibody (mouse monoclonal antibody) were purchased from local suppliers.

Wort solid medium was prepared by adding 2% agar powder to 5° wort, while seed medium contained 30 g L^-1^ corn flour, 50 g L^-1^ soybean flour, 1.5 g L^-1^ KH_2_PO_4_, 0.5 g L^-1^ MgSO_4_ 7H_2_O. YEP medium (pH7.0) was composed of 10 g L^-1^ beef extract, 10 g L^-1^ yeast extract, 5 g L^-1^ NaCl, while PD medium utilized 200 g L^-1^ potato, 20 g L^-1^ glucose, 1 g L^-1^ KH_2_PO_4_, 0.1 g L^-1^ MgSO_4_.7H_2_O.

### 2.2 Agrobacterium-mediated transformation of *Blakeslea trispora* protoplasts

An appropriate amount of *B. trispora* spore solution was spread on wort solid medium and incubated at 25 °C for 5 days. Some lawn was then scraped off and inoculated into seed medium, and cultivated in a shaker at 25 °C and 180 rpm for 60 h. The *B. trispora* culture was centrifuged at 8,000 rpm for 10 min, and the mycelia were washed twice with sterile water. The mycelia were then added to a previously prepared enzymatic hydrolysis solution (2% lysozyme, 0.5% lysozyme, 0.25% helicase and 0.25% cellulase dissolved in 0.6 M NaCl solution), and incubated at 28 °C in a shaker at 75 rpm for 4 h in darkness. After enzymatic hydrolysis, the *B. trispora* mycelia were filtered through three layers of lens-cleaning paper to collect protoplasts. The obtained protoplasts were washed with 0.6 M NaCl solution, centrifuged at 4,000 rpm for 10 min, and then diluted to 10^6^ mL^-1^ for later use.

The constructed binary vector was transformed into competent *A*. *tumefaciens* LBA4404 cells as follow. An appropriate amount of plasmid was added to 100 μL of competent cells, mixed gently, and placed on ice for 5 min, then in liquid nitrogen for 5 min, in a 37 °C water bath for 5 min, and finally on ice for 5 min. Subsequently, LB liquid medium was added and cells were incubated at 28 °C in a shaker for 2–3 h. The transformed cells were then spread on LB solid plates supplemented with 100 μg·mL^-1^ kanamycin and 50 μg·mL^-1^ rifampicin, and incubated at 28 °C until single colonies appeared.

The LBA4404 strain containing the target vector was cultured in 50 mL of YEP liquid medium until the OD_600_ reached 0.4–0.6. Then 400 μL of the activated-Agrobacterium-culture solution was mixed with 200 μL of *B. trispora* protoplasts. Acetosyringone was added to a final concentration of 200 μM, and the culture was incubated at 28 °C and 180 rpm in darkness for 48 h. After that, 800 μL of PD liquid medium was added, and incubation continued at 28 °C and 180 rpm for 24 h. Finally, 200 μL of the culture solution was spread on wort medium supplemented with 200 μg·mL^-1^ cephalosporin and 100 μg·mL^-1^ hygromycin or 75 μg·mL^-1^ geneticin, and cultured at 25 °C for about 3–4 days.

### 2.3 Construction of *btcrgA* promoter truncation mutants

Primers were designed to amplify the upstream sequence (approximately 2000 bp) of the *btcrgA*-transcription start site as the promoter region using the extracted *B. trispora* genome as a template. The potential promoter sequences were integrated into the pUC57 vector, transformed into *E*. *coli* DH5α and cultivated overnight. Colonies were picked and sequenced. Cis-regulatory elements in the *btcrgA* promoter sequence were investigated using PlantCARE, and possible transcription start sites were predicted using BDGP.

For the construction of *btcrgA* promoter truncation mutants, the *btcrgA* promoter sequence was gradually truncated from the 5′ end without disrupting the identified cis-regulatory elements, as shown in Supplementary Figure S1. The *btcrgA* promoter sequences of different lengths were amplified, and used to replace the original CaMV35S promoter, constructing pCambia1303-procrgA-F, F1, F2 and F3, respectively. The Hieff Clone®Plus Multi One Step Cloning Kit was used to ligate the fragments into the pCAMBIA-1303 vector, which was then transformed into competent *E*. *coli* DH5α. The plasmid was extracted and transformed into *A. tumefaciens* LBA4404. PCR amplification and sequencing confirmed that the constructed plasmid with the correct sequence was transferred into *Agrobacterium*, which was then used to infect *B. trispora*. Wild-type and transformed *B*. *trispora* were cultured on wort solid medium in darkness at 25 °C for 2 days, and then cultured under white light (15 W·m^-2^) or in continued darkness.

*Blakeslea trispora* transformants grown on wort medium were collected, washed with PBS solution, and centrifuged at 8,000 rpm for 10 min. The fresh mycelia were gained and ground thoroughly in a mortar with liquid nitrogen, and total protein was extracted according to the GUS detection kit instructions. GUS-mediated catalysis converts 4-methyl-7-acetoxycoumarin-β-D-glucuronide (4-MUG) to 4-methylumbelliferon (4-MU). The excitation and emission wavelengths of 4-MU are 356 nm and 456 nm, respectively. The absorbance of the samples at 456 nm was measured using a multifunctional microplate reader (synergy H4), and the concentration of 4-MU generated in each reaction was calculated using a standard curve.

### 2.4 Construction of target gene RNA interference and overexpression strains

Using the *B. trispora* genome as a template, the *btcrgA* and *btwc-1b* gene sequences were amplified by PCR and sequenced. Based on these sequences, interference sequences targeting *btcrgA* and *btwc-1b* were designed using BLOCK-iT™ RNAi Designer online tool. Supplementary Figure S2 shows a schematic diagram of the construction of overexpression and RNA interference (RNAi) vectors for *btwc-1b*. The construction procedure for *btcrgA* overexpression and RNAi vectors was similar, except for the target sequence.

The primer sequences for each shRNA are listed in Supplementary Table S1. The mouse U6 promoter was amplified from pmU6-gRNA and used as the promoter for expressing shRNA. pCAMBIA-2301 containing the mU6-shRNA sequence was integrated into the genome of *B. trispora* via *Agrobacterium*-mediated transformation to generate strains with specific gene interference. In the same way, overexpression strains CaMV35S-*btwc-1b* (or *btcrgA*) were constructed.

### 2.5 RNA extraction, reverse transcription, and RT-PCR

An appropriate amount of *B. trispora* mycelia was collected and ground thoroughly with liquid nitrogen. RNA was then extracted and purified according to the RNApure kit instructions. Agarose gel electrophoresis was used to assess the quality of the extracted RNA. Reverse transcription was performed using the Hifair® III first Strand cDNA Synthesis SuperMix for qPCR (gDNA digester plus) kit with the extracted RNA as template to obtain corresponding cDNA. The obtained cDNA and other components were added according to Heffi® qPCR SYBR Green Master Mix kit instructions, followed by detection using a real-time PCR instrument. The real-time quantitative PCR reaction program was as follows: pre-denaturation at 95 °C for 5 min, followed by 4 cycles of denaturation at 95 °C for 10 s and annealing/extension at 60 °C for 30 s. *tef1* was used as an internal reference gene, and the relative transcription level of each target gene was calculated using the 2^−ΔΔCt^ method.

### 2.6 Construction of recombinant strains for BiFC and GST-pull down experiments

The 5′ 1–519 bp sequence and 3′ 465–717 bp sequence of the Venus fluorescent protein were cloned to obtain Venus^N^ and Venus^C^ fragments, which were inserted between the CaMV35S promoter and the NOS terminator, respectively. A flexible linker (GGGGS)_3_ was inserted between *btcrgA* and Venus^N^ and between *btwc-1b* and Venus^C^ to obtain the recombinant construct pCambia2301-*btcrgA*-Venus^N^-*btwc-1b*-Venus^C^ (Supplementary Figure S3). The resulting *B. trispora* recombinant strain was washed with PBS solution and centrifuged at 8,000 rpm for 10 min, repeated three times. Some mycelia were then placed on slides, and fluorescent signals were observed using an inverted fluorescence microscope. Bimolecular fluorescence complementation (BiFC) works by fusing two non-fluorescent fragments of a fluorescent protein to two proteins of interest. When the two proteins interact in the cell, the fluorescent protein fragments come together to form a functional fluorophore, generating a fluorescent signal.

To express proteins required for the pull-down experiment, the pET28a-*btcrgA* plasmid maintained in our laboratory was used as the starting vector. The GST coding sequence was added to the 5′ end of the *btcrgA* sequence, linked by a flexible linker (GGGGS)_3_. The resulting vector, pET28a-GST-*btcrgA*, was constructed through homologous recombination for expression of the bait protein. The plasmid pET28a-*btwc*-*1b*, also from our laboratory stock, was used for expression of the target protein. Both plasmids were transferred into *E. coli* BL21 (DE3) for subsequent *in vitro* protein interaction assays.

### 2.7 Determination of β-carotene content

The mycelia were collected, quickly placed into centrifuge tubes wrapped in aluminum foil, washed three times with distilled water, and then dried in a vacuum drying oven at 45 °C for 48 h. The dried mycelia were ground to a powder followed by repeated extraction with absolute ethanol (containing 2% BHT) until the mycelia were colorless. β-Carotene was detected at 450 nm using a high-performance liquid chromatograph equipped with a Zorbax SB-C18 column, with acetonitrile (15%, v/v) as the mobile phase at a flow rate of 1.0 mL·min^-1^.

### 2.8 Correlation and significance analyses

Correlation analysis was used to determine the correlations between the contents of β-carotene and the transcription levels of carotenoid biosynthesis-related genes. The experimental data were all taken from the mean ± SD of three parallel samples. One-way analysis of variance by SPSS was conducted to determine the significance of the effect of *btcrgA* promoter sequence on GUS enzyme activity.

## 3 Results and discussion

### 3.1 Correlation analysis of transcription of *btcrgA*, *btwc-1b* and carotenoid structural genes

**FIGURE 1:**
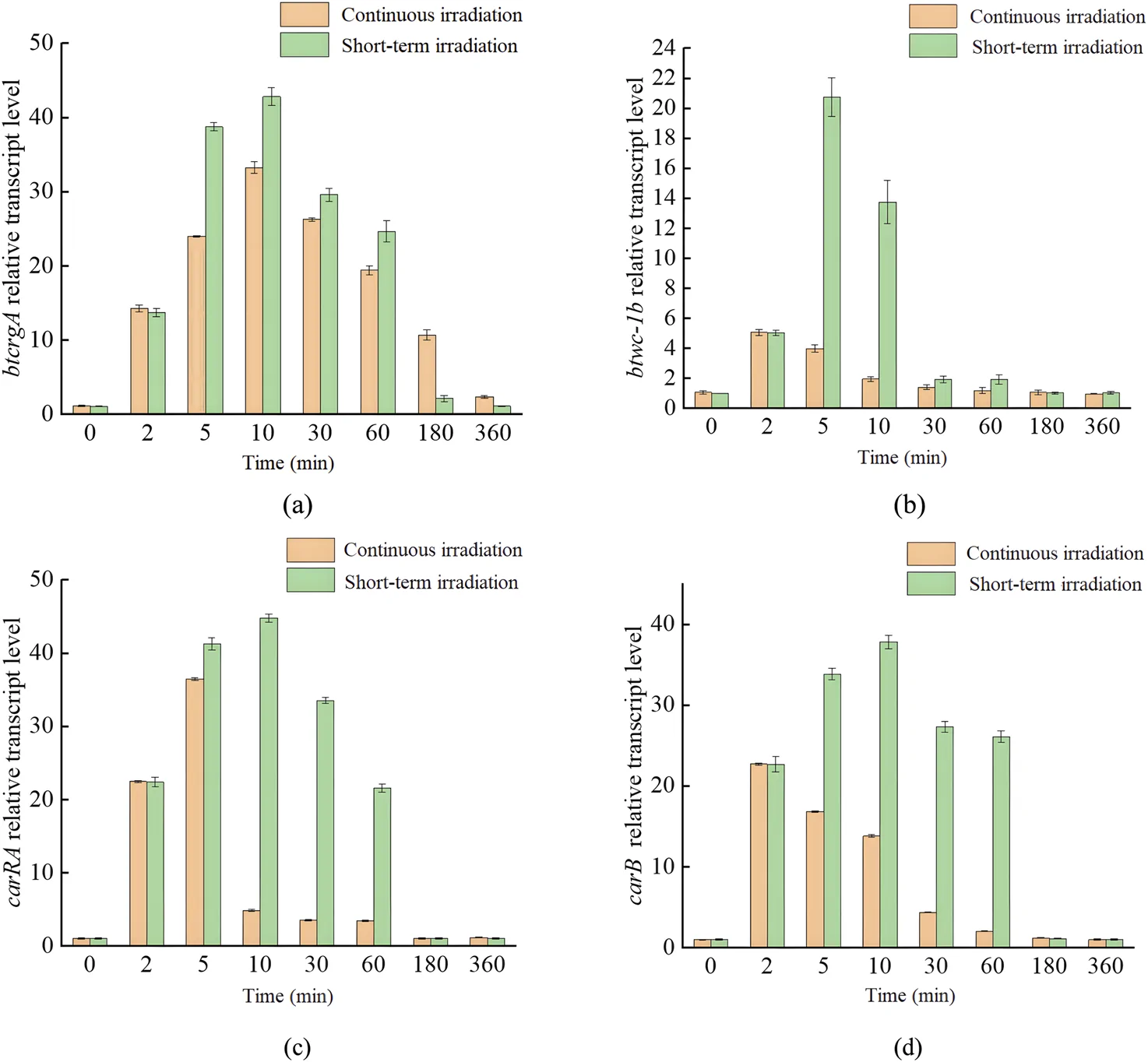
Relative transcription levels of each gene under continuous and short-term irradiation in *Blakeslea trispora*. **(a)** *btcrgA*; **(b)** *btwc-1b*; **(c)** *carRA*; **(d)** *carB*. All groups were pre-cultured in darkness for 2 days, and continuous irradiation denotes mycelia were then continuously exposed to light (15 W·m^-2^), while short-term irradiation indicates mycelia were exposed to light (15 W·m^-2^) for 2 min and then cultured in darkness.

The transcription levels of *btcrgA*, *btwc-1b*, *carRA* and *carB* were investigated by qPCR (Figure 1). Regardless of short-term or continuous irradiation, the transcription levels of *btcrgA*, *btwc-1b*, *carRA* and *carB* showed a rapid response, indicating that these genes are all “light-induced”. However, under continuous irradiation, the transcription level of each gene (except *btcrgA*) reached a maximum at 2–5 min and then decreased rapidly. In contrast, under short-term light exposure, their transcription levels generally remained elevated for the 10-min duration. Therefore, continuous irradiation produced an obvious photoinhibition effect, resulting in a rapid decrease in the transcription levels of these genes. In filamentous fungi, this reduction in gene transcription due to photoinhibition is termed “photoadaptation”, a phenomenon widely observed in various filamentous fungi such as *Ascomycetes* and *Zygomycetes* (Chen et al., 2010).

To investigate the relationship between the above genes in the regulation of carotenoid synthesis, a correlation analysis of their transcription levels was performed (Table 1). Under continuous irradiation, the correlation coefficients for *btcrgA*/*carRA btcrgA*/*carB* and *btcrgA*/β-carotene content were 0.976, 0.952 and 0.879, respectively, indicating a strong, statistically significant correlation with (P < 0.01). Apart from this, *btcrgA* showed no significant correlation with *btwc-1b*, and *btwc-1b* also displayed no significant correlation with the carotenoid structural genes (*carRA* and *carB*) and β-carotene content. On the other hand, the correlation coefficients for all bivariates were high under short-term irradiation, displaying significant or extremely significant correlation. Considering the photoinhibition effect caused by continuous irradiation, the significant correlation observed under short-term irradiation may better indicate that *btcrgA* and *btwc-1b* could interact in regulating carotenoid synthesis.

**TABLE 1:** Correlation analysis of the relative transcription levels of each gene under continuous and short-term irradiation.

Treatment	Variate	Spearman correlation coefficient				
		β-carotene content	*btcrgA*	*btwc-1b*	*carRA*	*carB*
Continuous irradiation	*btcrgA*	0.879***	1***	0.357	0.976***	0.952***
	*btwc-1b*	0.388	0.357	1***	0.286	0.429
	β-carotene content	1***	0.879***	0.388	0.953***	0.910***
Short-term irradiation	*btcrgA*	0.922***	1***	0.905***	0.952***	0.976***
	*btwc-1b*	0.885***	0.905***	1***	0.929***	0.881***
	β-carotene content	1***	0.922***	0.885***	0.973***	0.942***

***, ** and * represent the significance levels of 1%, 5%, and 10%, respectively.

### 3.2 Photoreresponsive activity of the *btcrgA* promoter

**FIGURE 2:**
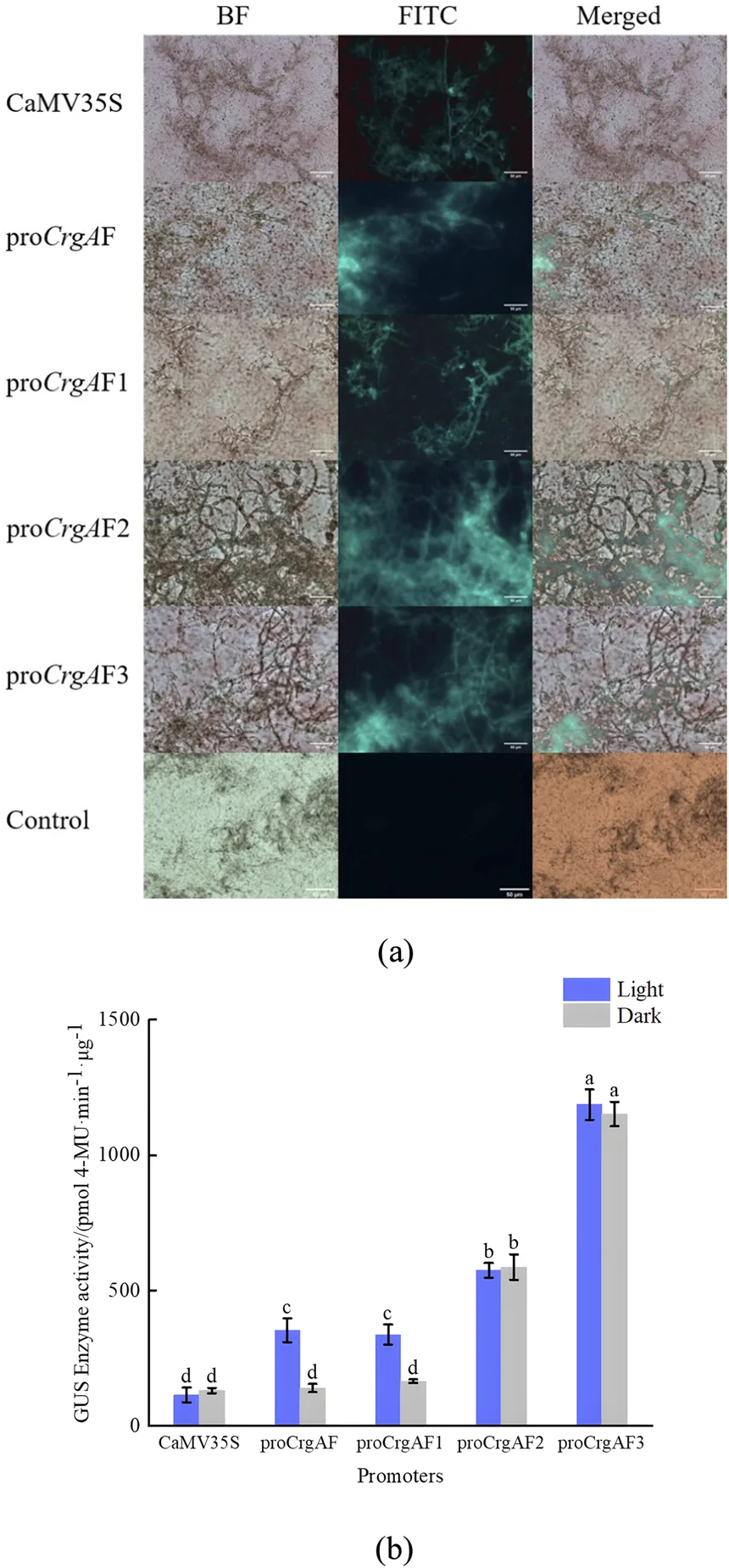
Activity analysis of *btcrgA* promoter sequence. **(a)** Fluorescence spectrum *Blakeslea trispora* mycelia due to the expression of mGFP5 driven by different truncated *btcrgA* promoters. The scale bar represents 50 μm. **(b)** Enzyme activities of GUS that expressed in *Blakeslea trispora* driven by different length promoters. The enzyme activity was defined as the ratio of 4-MU amount to total protein amount and time (4-MU·min^-1^·μg^-1^).

Figure 1a shows that the expression of *btcrgA* is regulated by light, which may be ascribed to the function of its promoter. Bioinformatics analysis indicated that multiple photoresponsive elements are present in the *btcrgA* promoter, such as Sp1, LAMP element, and TCT motif. (Supplementary Table S2). Figure 2a shows that there was no background expression of mGFP5 in untransformed *B. trispora* (control), while fluorescent signals were observed in the transformants. The CaMV35S promoter could drive the expression of the GUS-mGFP5 fusion protein in *B. trispora*, and all four truncated *btcrgA* promoters enabled foreign gene expression. GUS enzyme activities were then determined (Figure 2b). Wild-type *B. trispora* showed no catalytic activity towards 4-MUG, while strains carrying CaMV35S or the different truncated *btcrgA* promoters showed significant activity. Under light irradiation, the order of enzyme activity was pro*CrgA*F3 > pro*CrgA*F2 > pro*CrgA*F1 > CaMV35S. There was no significant difference in the ability of the pro*Crg*AF1 and pro*Crg*AF promoters to drive GUS expression, while pro*Crg*AF3 drove approximately twice the activity of pro*crg*AF2.

A significant difference in GUS enzyme activity between the pro*Crg*AF and pro*Crg*AF1 constructs was observed under dark vs. light conditions, suggesting the presence of a light responsive element within the 1,499–989 bp region of the promoter. Analysis of this promoter region revealed the presence of light-responsive elements such as AE Box, G Box and SP1. Furthermore, the strong activity of the pro*CrgA*F3 promoter makes it a potential candidate as an endogenous strong promoter for expressing exogenous genes in *B. trispora*, and provides a basis for fine-mapping activating and repressing elements within the *btcrgA* promoter.

### 3.3 Effects of *btcrgA* on β-carotene synthesis and its transcriptional regulation of carotenoid structural genes and *btwc-1b*

**FIGURE 3:**
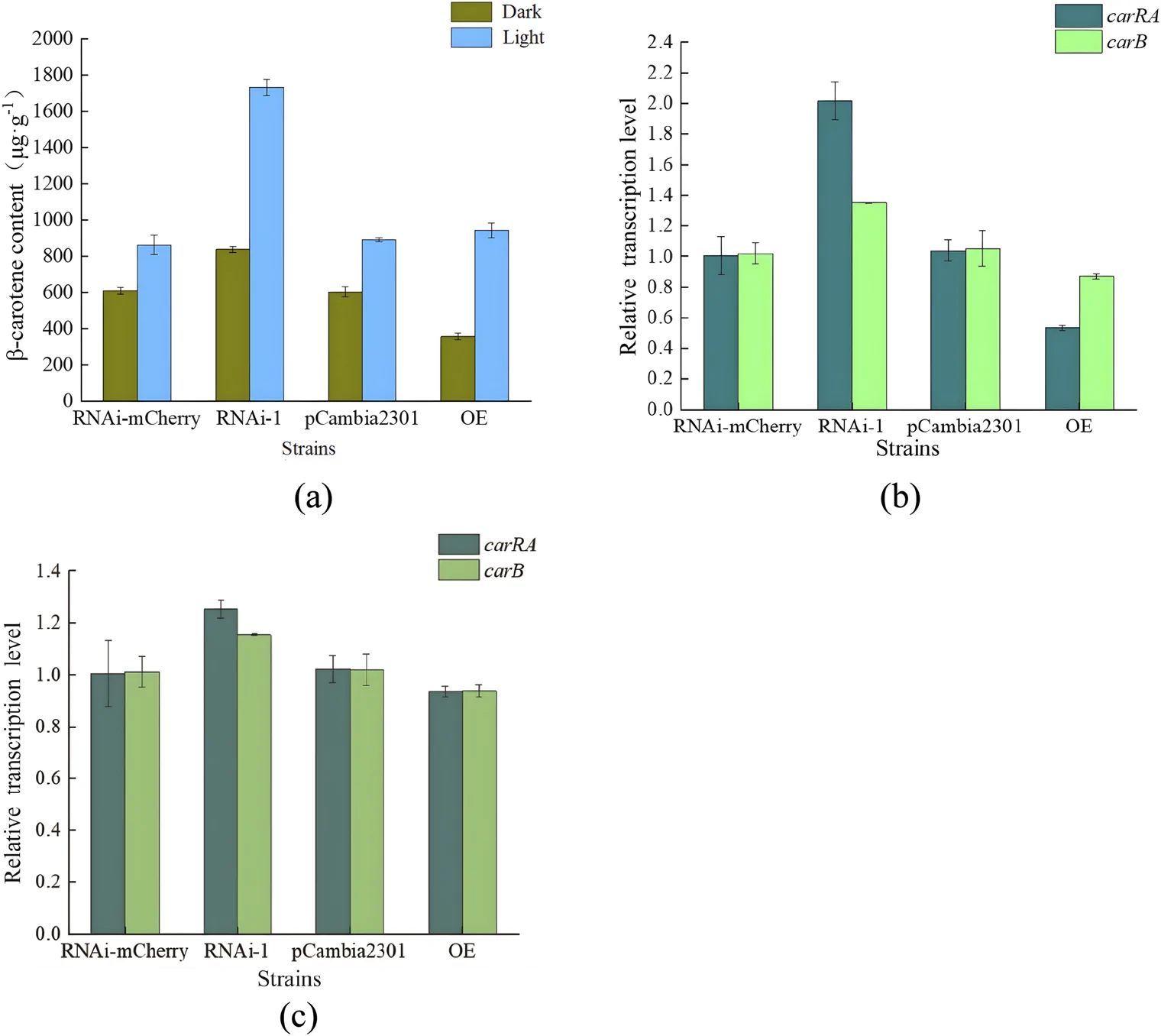
Effects of *btcrgA* on β-carotene synthesis and its regulation on carotenoid structural genes and *btwc-1b* transcription levels. Dark denotes that strains were cultured in darkness for 3 days, while light indicates that they were cultured in darkness for 2 days, then in light (15 W·m^-2^) for 1 day. **(a)** β-Carotene content in different *Blakeslea trispora* strains. RNAi-1 is the *btcrgA* interference strain (RNAi-mCherry was used as the control), and OE is the *btcrgA* overexpression strain (pCambia2301 was used as the control). Relative transcription levels of *carRA* and *carB* in *btcrgA* RNAi and overexpression strains in darkness **(b)** and light **(c)**.

Compared with the control strain, the β-carotene content in the *btcrgA*-RNAi strain increased by 37.4% in darkness, while it decreased by 41.4% in the overexpression strain (Figure 3a). Meanwhile, the β-carotene content in the RNAi strain increased by 101% under light irradiation. These results confirm that *btcrgA* is a negative regulator of the β-carotene synthesis pathway in *B. trispora* (Luo et al., 2020c). In *Mucor circinelloides*, overexpression of the full-length *crgA* sequence or a 3′-truncated version led to increased *crgA* mRNA levels under both dark and light conditions and a clear accumulation of β-carotene (Navarro et al., 2000). This differs from our results, where overexpression of *btcrgA* in *B. trispora* did not significantly alter β-carotene under light irradiation.

To understand whether changes in carotenoid synthesis are related to transcription, the transcription levels of carotenoid structural genes in the different strains were determined. Figure 3b shows that the relative transcription levels of *carRA* in the RNAi-1 and overexpression strains were 2.0 and 0.53, respectively, and those of *carB* were 1.35 and 0.87 in darkness. Under light irradiation, the relative transcription levels of *carRA* were 1.25 and 0.93, and those of *carB* were 1.15 and 0.94 (Figure 3c). These results suggest that the effect of *btcrgA* on β-carotene synthesis is related to transcriptional changes in the carotenoid structural genes. However, the relative transcription level of the *btwc-1b* did not change significantly (data not shown), indicating that *btcrgA* does not affect *btwc-1b* at the transcriptional level.

### 3.4 Effects of *btwc-1b* on β-carotene synthesis and its transcriptional regulation of carotenoid structural genes and *btcrgA*

**FIGURE 4:**
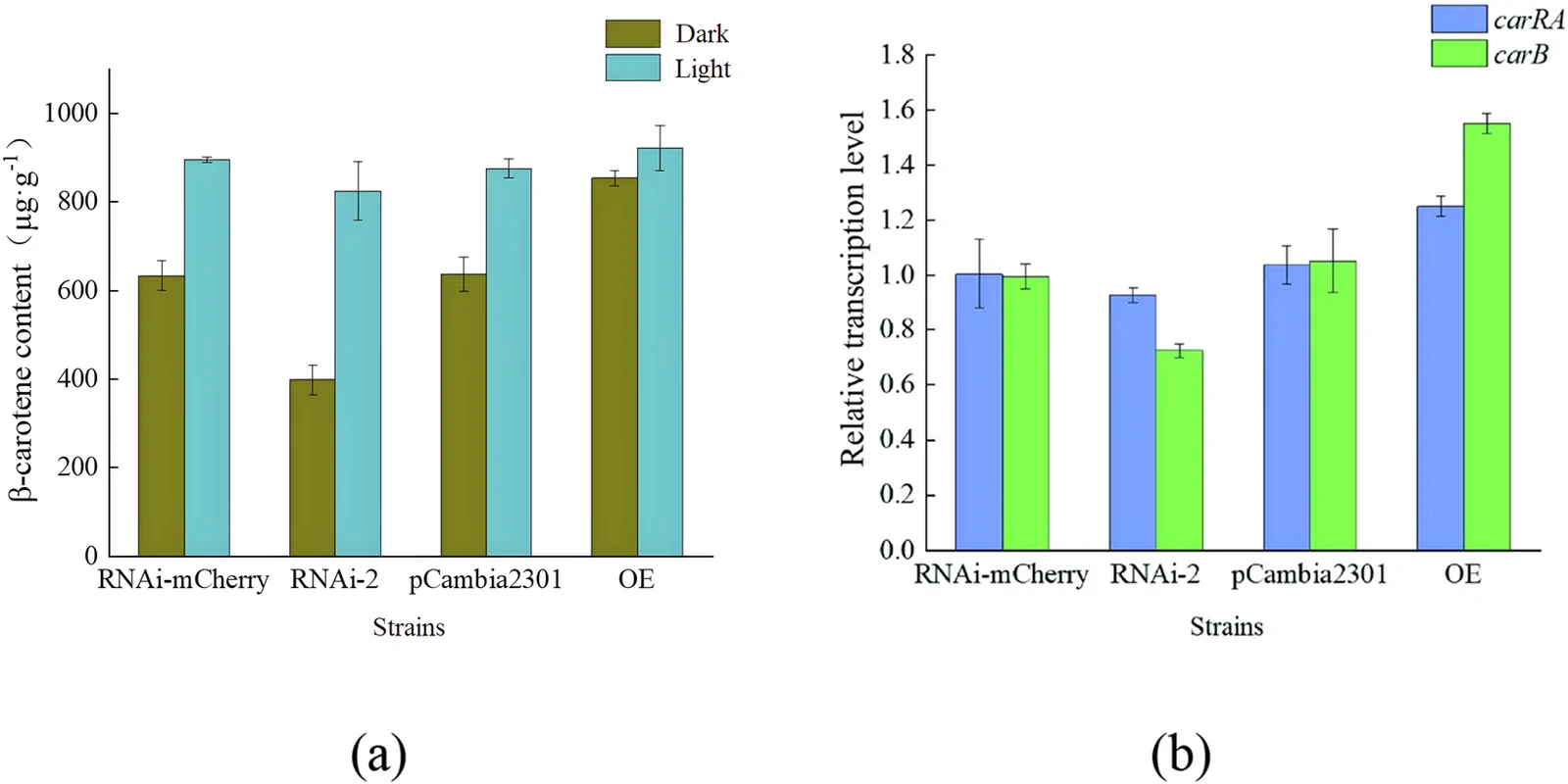
Effects of *btwc-1b* on *Blakeslea trispora* β-carotene synthesis and its regulation on carotenoid structural genes and *btcrgA* transcription levels. Dark denotes that strains were cultured in darkness for 3 days, while light indicates that they were cultured in darkness for 2 days, then in light (15 W·m^-2^) for 1 day. **(a)** β-Carotene content in different *Blakeslea trispora* strains. RNAi-2 is the *btwc-1b* interference strain (RNAi-mCherry was used as the control), and OE is the *btwc-1b* overexpression strain (pCambia2301 was used as the control). **(b)** Relative transcription levels of *carRA* and *carB* in *btwc-1b* RNAi and overexpression strains in darkness.

β-Carotene content was extracted and quantified for each strain. Accumulation of β-carotene in the *btwc-1b*-RNAi and overexpression strains was 400 μg·g^-1^ and 854 μg·g^-1^, respectively (Figure 4a). Those values are much higher or lower than that in the control group (630 μg·g^-1^), indicating that accumulation of β-carotene is affected by *btwc-1b* in darkness. However, the β-carotene contents in *btwc-1b* RNAi and overexpression strains were not significantly different from the control group under light irradiation, suggesting that light abolishes the regulatory effect of *btwc-1b* on β-carotene biosynthesis.

Futhermore, the effect of *btwc-1b* on β-carotene synthesis was investigated by determining the relative transcription levels of *carRA* and *carB* in RNAi and overexpression strains. Figure 4b shows that the relative transcription level of *carRA* was 0.92 in the *btwc-1b* RNAi strains, while that of *carB* was reduced to 0.73. Conversely, these values for *carRA* and *carB* increased to 1.25 and 1.55 in the *btwc-1b*-overexpression strains, respectively. Therefore, *btwc-1b* can affect the expression of carotenoid structural genes in *B. trispora* in darkness. However, light appeared to eliminate this regulation, as there were no significant differences in the relative transcription levels of *carRA* and *carB* under light irradiation (data not shown). Furthermore, qPCR analysis showed that *btwc-1b* did not regulate the expression of *btcrgA* at the transcriptional level under continuous light irradiation (data not shown).

The accumulation of β-carotene in an *M. circinelloides* Δ*wc-1b* strain and the wild-type strain is similar and there was no significant difference in the amount of *carRA* and *carB* mRNA detected by agarose gel electrophoresis (Silva et al., 2006), suggesting that *mcwc-1b* may not be involved in the light-induced regulation of β-carotene synthesis in *M. circinelloides*. In this study, the relative transcription levels of *carRA* and *carB* in *btwc-1b* RNAi and overexpression strains cultured under continuous irradiation did not change compared to the control strains, and β-carotene accumulation was also similar. However, the relative transcription levels of *carRA* and *carB* in the RNAi and overexpression strains cultured in darkness differed from the pattern observed in the *M. circinelloides* Δ*wc-1b* strain. Thus, *btwc-1b* can regulate the expression of carotenoid structural genes and β-carotene accumulation in darkness, while its involvement in the regulation of light-induced β-carotene synthesis remains unclear.

### 3.5 Protein interaction between BtCrgA and BtWC-1b

**FIGURE 5:**
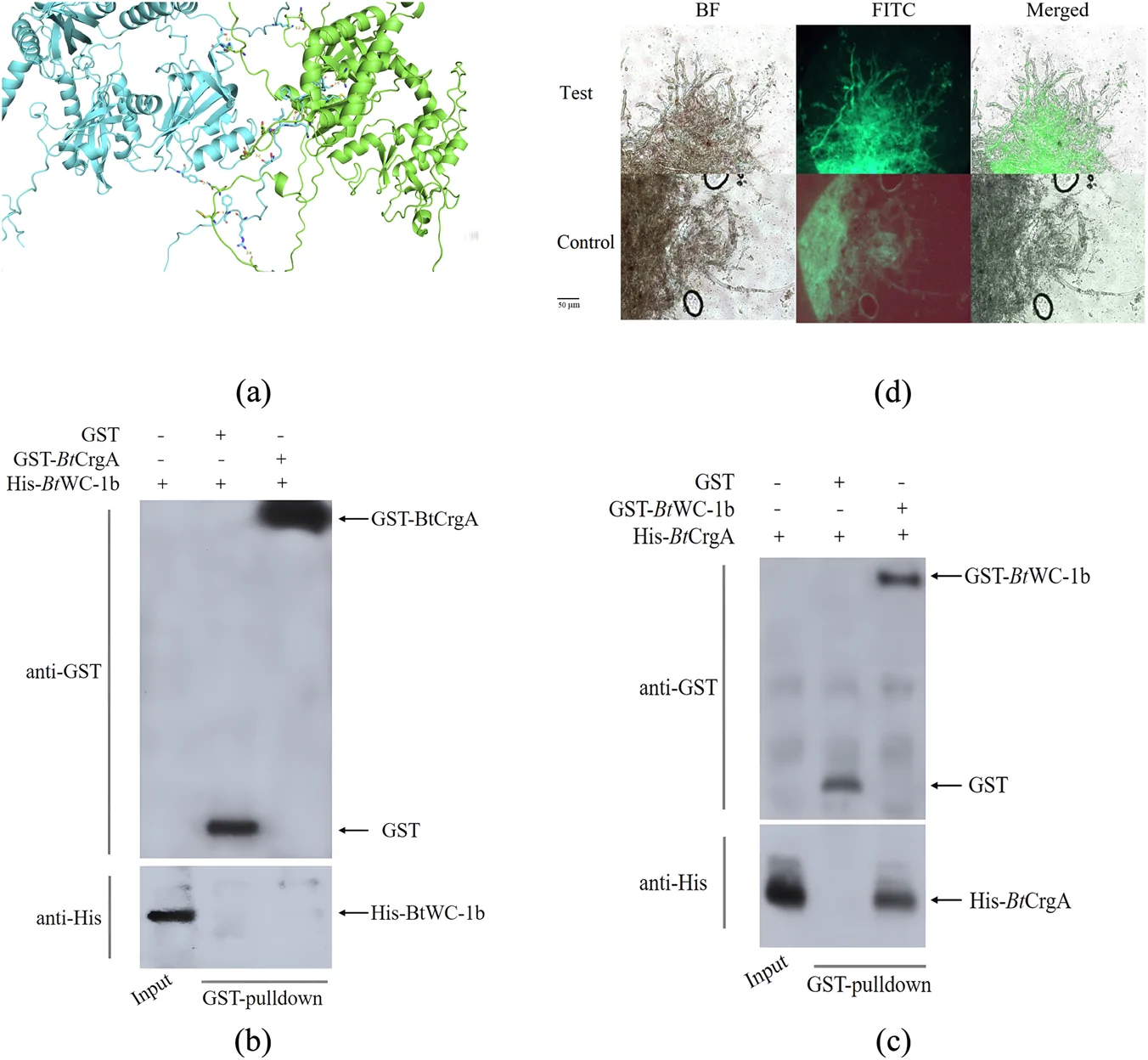
Protein interaction between BtCrgA and BtWC-1b. **(a)** BtCrgA and BtWC-1b protein interaction prediction model. Green, blue and yellow parts denote BtCrgA, BtWC-1b and hydrogen bond, respectively. **(b)** GST-BtCrgA interacts with His-BtWC-1b *in vitro*. **(c)** GST-pull down GST-BtWC-1b interacts with His-BtCrgA *in vitro*. **(d)** Observation of Venus protein fluorescence signal. The scale bar represents 50 μm. BF: bright field, FITC: laser wavelength is 490–495 nm, Merged: BF and FITC fusion image.

Subcellular localization of BtCrgA and BtWC-1b was predicted using the WoLF PSORT and Cell-PLoc 2.0 online tools, which suggested that BtCrgA localizes to the nucleus and BtWC-1b localizes to both the cytoplasm and nucleus. Potential multimeric structures of the CrgA and WC-1b proteins were predicted using ZDOCK v3.0.2 (Seeliger and de Groot, 2010; Pierce et al., 2011; Kastritis et al., 2014; Pierce et al., 2014; Xue et al., 2016), and the top ten high-scoring polymer structures were listed. PRODIGY v2.0 was then used to analyze the predicted multimer structure, and obtain the binding affinity and dissociation constant for the CrgA-WC-1b complex (Kastritis et al., 2014; Vangone and Bonvin, 2015; Xue et al., 2016). Figure 5a indicates predicted strong binding between BtCrgA and BtWC-1b, with a dissociation constant of 7.5 e^−17^. Amino acid pairs predicted to form hydrogen bonds include 224Y-1M, 252Q-275K, 416A-27R, 421M-29F, 424N-123Y, 458Q-232P, 458Q-233N, 521S-11F, 551N-6S, 593N-14Q, 597T-15I and 599S-18S.

To verify the reliability of this prediction, *in vitro* interaction analysis was performed using a GST-pull down strategy. Purified GST-tagged BtCrgA and GST-tagged BtWC-1b were used as bait proteins, with His-tagged WC-1b and His-tagged BtCrgA were used as target proteins, respectively. Figure 5b shows that a GST monoclonal antibody captured the GST-BtCrgA protein in lane 3, but a His monoclonal antibody did not detect any signal, indicating no binding between GST-BtCrgA and His-BtWC-1b under these conditions. The lack of interaction *in vitro* between GST-BtCrgA and His-BtWC-1b might be ascribed to the possibility that N-terminal GST tag on BtCrgA interferes with its recognition of a degron sequence at the N- or C-terminus of BtWC-1b (Sherpa et al., 2022). To test this hypothesis, the tags on the two proteins were swapped. In subsequent experiments, a His monoclonal antibody captured His-BtCrgA (Figure 5c), indicating that GST-BtWC-1b can interact with His-BtCrgA *in vitro*.

Furthermore, *in vivo* interaction was analyzed using a BiFC strategy. Fragments encoding Venus^N^-Venus^c^ (control) and *btcrgA*-VenusN-*btwc-1b*-Venus^C^ fragments were integrated into the *B. trispora* genome. Mycelia of the verified strains were observed under a fluorescence inverted microscope. As shown in Figure 5d, the experimental group showed clear fluorescent signals, while the control group showed only minimal fluorescence, even after increasing exposure time. Under the same exposure settings, the control showed almost no fluorescence. These results indicate that BtWC-1b and BtCrgA are in close proximity and interact with each other *in vivo*.

BtCrgA possesses a RING-finger domain, suggesting it may function as an ubiquitin ligase. Our research team has previously isolated and identified one ubiquitin activating enzyme (BtE1) and 18 putative ubiquitin-conjugating enzymes (BtUBC) from *B*. *trispora*. BtE1, six BtUBC proteins, BtCrgA and BtWC-1b, were obtained through heterologous expression and affinity purification, and subjected to *in vitro* ubiquitination assays; However, BtCrgA could only ubiquitinate itself, not BtWC-1b ^[17]^. A possible reason is that the predicted and expressed BtUBC proteins used in this study are not the specific ubiquitin-conjugating enzymes enzyme required for WC-1b ubiquitination, or that the precise conditions required for the *in vivo* ubiquitination reaction could not be fully replicated *in vitro*.

## 4 Conclusion

The relative transcription levels of *btwc-1b*, *carRA*, and *carB* were highly correlated with that of *btcrgA* under short-term irradation conditions. Considering that both *btcrgA* and *btwc-1b* regulate β-carotene accumulation, it is reasonable to hypothesize that they may interact as signal transducers in light-induced β-carotene biosynthesis. The *crgA* promoter contains light-responsive elements, and its light-dependent regulatory effect was confirmed by truncation experiments.

*btcrgA* regulates the expression of carotenoid structural genes under both light and dark conditions, confirming its role as a negative regulator of carotene synthesis. Conversely, *btwc-1b* positively regulates β-carotene synthesis in darkness. However, light appears to counteract this regulatory effect. Although both genes play regulatory roles at the transcriptional level of carotenoid structural genes, no interaction between them was detected at this level.

Furthermore, at the protein level, we confirmed the interaction between BtCrgA and BtWC-1b through protein model prediction, *in vitro* pull-down and *in vivo* BiFC analyses. However, it remains unclear whether this interaction occurs through direct contact or is mediated by other proteins, perhaps in the context of an ubiquitination-related complex.

## Data Availability

The raw data supporting the conclusions of this article will be made available by the authors, without undue reservation.
