# Correction: Interaction between *crgA* and *wc-1b* in carotenoid synthesis in *Blakeslea trispora*

**DOI:** 10.3389/fbioe.2026.1992390

**Published:** 2026-09-23

**Authors:** Youai Dai, Renyue Gui, Jingyu Chen, Wei Luo

**Affiliations:** 1 Wuxi School of Medicine, Jiangnan University, Wuxi, China; 2 The Affiliated Wuxi People’s Hospital of Nanjing Medical University, Wuxi People’s Hospital, Wuxi Medical Center, Nanjing Medical University, Wuxi, Jiangsu, China; 3 The Key Laboratory of Industrial Biotechnology, Ministry of Education, School of Biotechnology, Jiangnan University, Wuxi, China

**Keywords:** *Blakeslea trispora*, BtCrgA, BtWC-1b, Protein interaction, RNA interference

Affiliation The Affiliated Wuxi People’s Hospital of Nanjing Medical University, Wuxi People’s Hospital, Wuxi Medical Center, Nanjing Medical University, Wuxi, Jiangsu, China was omitted from the paper. This affiliation has now been added for author(s) Youai Dai and Jingyu Chen.

The original version of this article has been updated.

