# Supplementary material for: Interaction between crgA and wc-1b in carotenoid synthesis in Blakeslea trispora
Source: Front Bioeng Biotechnol. 2026 Jul 28;14:1838537. doi: 10.3389/fbioe.2026.1838537 (PMC13457194; doi:10.3389/fbioe.2026.1838537)
Supplement: Supplementary file 1 [file Supplementaryfile1.docx]

Table S1 The RNAi target site sequences of *crgA and wc-1b*.

| Target site | Target site sequence (5′-3′) | GC value |
| --- | --- | --- |
| *btwc-1b*-1 | CAGTCGATACTCTGGCATG | 53% |
| *btwc-1b*-2 | GTCCAGTCGACTTATCATG | 47% |
| *btwc-1b*-3 | GTGGTGGATGCTAAACAAT | 42% |
| *btcrgA*-1 | CAAAGCATACAACAACATA | 32% |
| *btcrgA*-2 | CACTTTACCTGTAGGACAA | 42% |
| *btcrgA*-3 | TGTCATGGCAAATTGAATA | 32% |

Table S2 Putative photoresponsive elements and their positions in the *btcrgA* promoter.

| Name of cis-acting element | Sequence (5′-3′) | Function | Location | Number |
| --- | --- | --- | --- | --- |
| AE-box | AGAAACAA | part of a module for light response | 595(+) | 1 |
| Box4 | ATTAAT | part of a conserved DNA module involved in light responsiveness | 1459(+) | 1 |
| G-box | CACGTC | involved in light responsiveness | 956(+) | 1 |
| LAMP-element | CTTTATCA | part of a light responsive element | 158(+) | 1 |
| TCT-motif | TCTTAC | part of a light responsive element | 627(-) | 1 |
| Sp1 | GGGCGG | light responsive element | 870(+), 1370(+) | 2 |

Fig. S1 Truncated mutation of *btcrgA* promoter fragment and recombinant vector framework. (a) Pro*crgA*-Fs with different truncation lengths. (b) The position structure diagram of the *btcrgA* promoter replacing the CaMV35S promoter.

Fig. S2 Frameworks of overexpression and RNA interference vectors for *btwc-1b*.

Fig. S3 Vector frameworks for BiFC experiment.
